# Impact of early rehabilitation therapy on functional outcomes in patients post distal radius fracture surgery: a systematic review and meta-analysis

**DOI:** 10.1186/s12891-024-07317-0

**Published:** 2024-03-05

**Authors:** Zhihui Zhou, Xiuying Li, Xiaoyan Wu, Xiaotian Wang

**Affiliations:** 1 Department of Emergency, Dongguan Hospital of Traditional Chinese Medicine, No. 3 Dongcheng Section, Songshan Lake Avenue, Dongcheng Street, Dongguan, Guangdong, 523000 China; 2 Traditional Chinese Medicine Department, Dongguan Songshan Lake Community Health Service Center, Lanxin Garden, Science and Technology, 9Th Road, Dongguan, Guangdong, 523000 China; 3 Department of Hand Surgery, Dongguan Hospital of Traditional Chinese Medicine, No. 3 Dongcheng Section, Songshan Lake Avenue, Dongcheng Street, Dongguan, Guangdong, 523000 China

**Keywords:** Distal radius fractures, Early rehabilitation, Meta-analysis, Upper limb function, Wrist function, Back extension mobility, Postoperative care

## Abstract

**Background:**

This meta-analysis aims to investigate the efficacy of early rehabilitation on patients who have undergone surgery for distal radius fractures (DRFs) with palmar plating, focusing on multiple outcome measures including upper limb function, wrist function, back extension mobility, pain levels, and complications.

**Methods:**

A rigorous search strategy adhering to the PRISMA guidelines was employed across four major databases, including PubMed, Embase, Web of Science, and the Cochrane Library. Studies were included based on stringent criteria, and data extraction was performed independently by two reviewers. Meta-analysis was conducted employing both fixed-effect and random-effects models as dictated by heterogeneity, assessed by the I^2^ statistic and chi-square tests. A total of 7 studies, encompassing diverse demographic groups and timelines, were included for the final analysis.

**Results:**

The meta-analysis disclosed that early rehabilitation yielded a statistically significant improvement in upper limb function (SMD -0.27; 95% CI -0.48 to -0.07; *P* < 0.0001) and back extension mobility (SMD 0.26; 95% CI 0.04 to 0.48; *P* = 0.021). A notable reduction in pain levels was observed in the early rehabilitation group (SMD -0.28; 95% CI -0.53 to -0.02; *P* = 0.03). However, there were no significant differences in wrist function (SMD -0.13; 95% CI -0.38 to 0.12; *P* = 0.36) and complications (OR 0.99; 95% CI 0.61 to 1.61; *P* = 0.96).

**Conclusions:**

Early rehabilitation post-DRF surgery with palmar plating has been found to be beneficial in enhancing upper limb functionality and back extension mobility, and in reducing pain levels. Nevertheless, no significant impact was observed regarding wrist function and complications.

##  Background

Distal radius fractures (DRFs), defined as fractures occurring within 3 cm of the articular surface of the radius, constitute one of the most common orthopedic injuries, displaying a significant clinical prevalence [1]. These fractures exhibit a unique bimodal age distribution, affecting both the elderly, often resulting from low-energy trauma like falls, and the pediatric population, where high-energy trauma is a common cause [2, 3]. This demographic duality complicates the clinical approach, necessitating a multi-faceted understanding of DRF management. The management of DRF is indeed complex and multifaceted, reflecting the diversity of the condition's etiology and presentation across various age groups. Historically, the standard treatment for DRF has primarily consisted of manual reduction followed by cast immobilization. This non-surgical approach was particularly common for non-displaced or minimally displaced fractures. With advancements in medical technology and surgical techniques, various options like external fixation, percutaneous pinning, and palmar plating have gained prominence [4]. Each method has its benefits and considerations, depending on factors such as fracture type, patient age, comorbidities, and surgeon expertise.

Early rehabilitation therapy following DRF surgery is pivotal in achieving complete functional recovery. It encompasses the prevention of complications such as joint adhesion and stiffness through timely interventions. Pain management strategies employ methods like cryotherapy and electrical stimulation to enhance comfort. Focus on mobility and function through strengthening and stretching exercises is essential to restore normal activities. Individualized planning tailors rehabilitation programs to specific needs, considering factors like fracture type and surgical method. Interdisciplinary collaboration among healthcare professionals fosters a cohesive care plan, aligning therapeutic goals with patient needs. Adherence to an evidence-based approach ensures that practices are informed by the latest scientific research, enhancing the efficiency and effectiveness of care delivery.

Despite the recognized benefits of early rehabilitation, conflicting opinions persist regarding the optimal timing, methods, and intensity of rehabilitation interventions post-DRF surgery. Varying practices across different clinical settings and lack of standardized protocols contribute to these discrepancies. This discordance underscores the pressing need for evidence-based guidelines that can inform clinical practice. Given this landscape, there exists a vital need to examine the current evidence on early rehabilitation therapy's impact on functional outcomes in patients following DRF surgery. This systematic review and meta-analysis aim to synthesize the available scientific literature, both nationally and internationally, on the subject. By conducting a rigorous evaluation of existing research, this paper seeks to identify best practices, highlight areas of uncertainty, and provide evidence-based recommendations.

## Methods

### Search strategy

In the execution of our meta-analysis, we rigorously adhered to the Preferred Reporting Items for Systematic Reviews and Meta-Analyses (PRISMA) guidelines, ensuring the consistency and transparency of our systematic review process and subsequent reporting [5]. The comprehensive literature search was conducted on June 25, 2023, across four major electronic databases: PubMed, Embase, Web of Science, and the Cochrane Library, without imposing any time restrictions. Search strategies were meticulously tailored, with specific adjustments in vocabulary and syntax to accommodate the unique requirements and structure of each individual database. The specific search terms of PubMed were: ("Distal Radius Fracture" OR "Distal Radius Fractures" OR "Radius Fracture, Distal"[MeSH Terms] OR "DRF") AND ("Postoperative" OR "Postoperative Care" OR "Post-Surgery" OR "After Surgery" OR "Postoperative Period"[MeSH Terms]) AND ("Early Rehabilitation" OR "Rehabilitation, Early" OR "Early Physical Therapy" OR "Early Intervention (Rehabilitation)"[MeSH Terms]) AND ("Wrist Function" OR "Wrist Mobility" OR "Wrist Joint Function" OR "Wrist Movement" OR "Range of Motion, Articular"[MeSH Terms]). Language constraints were not imposed during the search process. Additionally, the reference lists of pertinent studies were meticulously examined to identify any further records that could potentially contribute to the analysis.

### Inclusion criteria

Studies to be included in this Meta-analysis must fulfill the following criteria: 1) The participants should be patients aged 18 years and above who have undergone surgery for radial fractures, specifically distal radius fracture (DRF) fixation with palmar plating; 2) The early rehabilitation group must begin treatment within 2 weeks post-surgery, and the control group within 6 weeks, with a follow-up period of 3 months or more; 3) Intervention measures must include an early rehabilitation group identified as those receiving passive or active rehabilitation treatment immediately post-DRF surgery, and control group patients initiating functional exercises 2 to 6 weeks after fixation; 4) Outcome measures should include assessments of upper limb function, wrist function, dorsiflexion, flexion, pain, grip strength (kg), and complications.

The exclusion criteria were as follows: 1) Patients with concomitant tendon, vascular, or nerve injuries; 2) Studies with re-fractures at the same site or fractures accompanied by dislocation; 3) Documents with poor quality and lack of original data; 4) Case reports, commentaries, expert opinion and narrative reviews.

### Data extraction

The process of literature screening and data extraction for the Meta-analysis will be conducted by two independent evaluators, working independently but cross-referencing their work to ensure consistency. Should any discrepancies arise during this process, the involved reviewers will engage in a discussion to resolve the issue, and, if necessary, a third reviewer may be consulted for adjudication. The extracted data will encompass several categories: (1) Basic information such as the publication date, authors, country of origin, and age; (2) Details concerning the study subjects, including the type of study, surgical methods, time of rehabilitation intervention, the number of patients followed and the duration of follow-up, detailed descriptions of the treatments in both groups, and primary outcomes; and (3) For binary variables, the extracted data will include the number of cases, while for other continuous variables, the mean (Mean) and standard deviation (SD) will be extracted. In instances where the required data is not available in the published report, the investigators of the original study will be contacted via email to request the unpublished data.

### Quality assessment

The assessment of the quality of the studies included in the Meta-analysis was carried out utilizing the Cochrane Collaboration's risk of bias tool [6]. Two independent reviewers scrutinized specific domains: random sequence generation, allocation concealment, blinding of both participants and personnel, handling of incomplete outcome data, the possibility of selective reporting, and other potential bias-inducing factors. Each of these domains was evaluated and categorized as exhibiting either a low, unclear, or high risk of bias. In instances where disagreements between the reviewers occurred, a resolution was sought through thorough discussion, and, if required, consultation with a third reviewer was undertaken.

### Statistical analyses

The assessment of heterogeneity among the included studies was conducted utilizing chi-square statistics, with the degree of inconsistency quantified by the I^2^ statistic. An I^2^ value below 50% and an associated *P*-value equal to or greater than 0.10 were indicative of no substantial heterogeneity, thereby warranting the application of a fixed-effect model for the determination of the aggregated effect size. Conversely, the presence of significant heterogeneity was signified by an I^2^ value of 50% or higher, or a corresponding *P*-value less than 0.10, necessitating the employment of a random-effects model for calculating the combined effect size. In cases of pronounced statistical heterogeneity, further exploration was conducted through subgroup or sensitivity analysis to identify and mitigate potential sources of inconsistency. Sensitivity analysis was undertaken to assess the stability of the findings and to detect the individual influence of specific studies on the collective effect size, which entailed the sequential exclusion of each study followed by the recalculation of the overall effect measure. Evaluation of potential publication bias was accomplished through the examination of the funnel plot's symmetry, with an even distribution of data points around the apex indicating a diminished likelihood of influence by publication bias. Moreover, the Egger's linear regression test was implemented as a numerical method for detecting publication bias. All statistical evaluations were conducted as two-sided tests, with a *P*-value threshold of less than 0.05 constituting statistical significance. Analyses were performed utilizing Stata version 17 (StataCorp, College Station, TX, USA), ensuring methodological rigor and the validity of the conclusions derived from the Meta-analysis.

## Results

### Search results and study selection

**Fig. 1:**
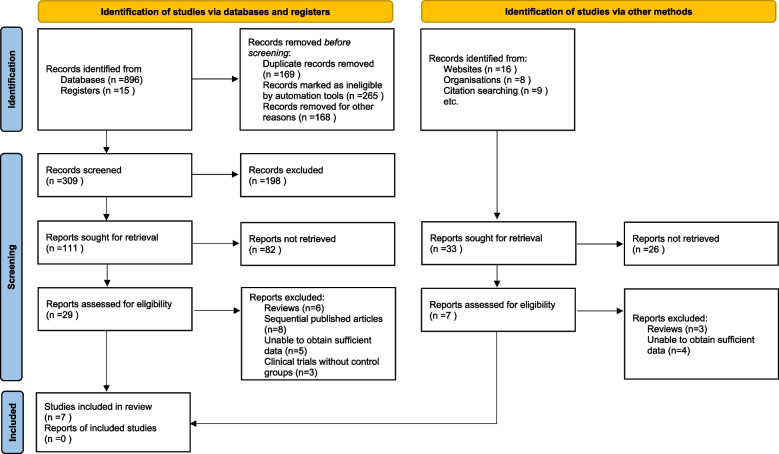
Selection process of included studies

In the preliminary search conducted across various electronic databases, a total of 944 pertinent articles were initially identified. Following the removal of duplicate entries and a careful examination of titles and abstracts, 29 relevant literatures were shortlisted, aligning with the predefined inclusion and exclusion criteria. Subsequent rigorous evaluation led to the exclusion of 22 articles upon detailed reading. Ultimately, a selection of 7 articles met the criteria and were included in the study [7–13]. A comprehensive visual representation of the literature screening process and outcomes is provided in Fig. 1, delineating the methodical progression from the initial discovery to the final inclusion.

### Study characteristics

The meta-analysis encompasses a total of seven studies, published between 2008 and 2020. The included studies engaged varying numbers of subjects in both early intervention and control groups, with the numbers ranging from 15 to 57 and 13 to 62 respectively. The age of participants across the studies ranged from a mean of 45.25 to 57 years in the early intervention groups and 49 to 58.77 years in the control groups, with some studies providing a range. Recovery times were specified differently across the studies, varying from immediate intervention to a span of 2 weeks. The follow-up periods ranged from 3 months to 2 years. This collective analysis aids in examining the variations in recovery time and outcomes in relation to different intervention strategies and methodologies, encompassing a diverse demographic and timeline (Table 1).

**Table 1:** Characteristics of studies included in the meta-analysis

Author	Year	Study Type	Early Intervention (n)	Control Group (n)	M/F (Early Intervention)	M/F (Control Group)	Age [years, Mean (Range)] (Early Intervention)	Age [years, Mean (Range)] (Control Group)	Recovery Time (Early Intervention)	Recovery Time (Control Group)	Follow-up Time
Calderón	2008	Prospective RCT	30	30	11/19	10/20	55	51	2 weeks	6 weeks	6 months
Brehmer	2014	Prospective RCT	36	42	NR	NR	49.8	55.3	2 weeks	4 weeks	6 months
Iitsuka	2016	Retrospective cohort	27	18	7/20	8/10	57 ± 13 (33 ~ 78)	49 ± 19 (19 ~ 81)	3 days	2 weeks	3 months
Quadlbauer	2017	Prospective RCT	15	13	2/13	2/11	49.13 ± 15.41	58.77 ± 12.06	Immediate	NR	1 year
Dennison	2018	Prospective RCT	18	15	7/23	4/26	54.9	53.1	2 weeks	5 weeks	1 year
Clementsen	2019	Prospective RCT	57	62	4/53	7/55	55 (12.4)	55 (11.9)	Immediate	2 weeks	2 years
Tomruk	2020	Prospective RCT	15	17	8/7	6/11	45.25 ± 9.84	57.00 ± 15.84	1 week	1 week	3 months

*RCT* Randomized controlled trial, *NR* Not report

### Results of quality assessment

**Fig. 2:**
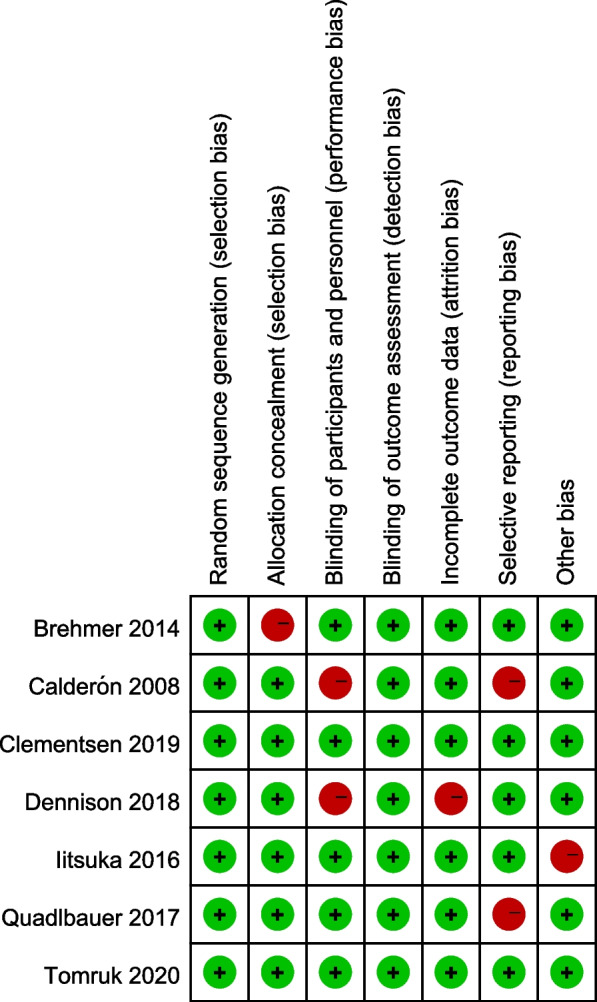
Quality assessment of included studies using Cochrane Collaboration's tool criteria. Red in figure indicates high risk, and green means low risk

An assessment of the risk of bias was meticulously executed across various domains within the seven incorporated studies. Among these, one investigation manifested a negligible risk of bias in all designated categories, reflecting an elevated degree of methodological precision and integrity. Conversely, a substantial risk of bias, constituting 28% of the studies, was identified within the realm of blinding of both participants and involved personnel. This unveils a conceivable influence of performance bias, potentially affecting the study outcomes in these instances. In a parallel finding, 28% of the encompassed randomized controlled trials exhibited a pronounced risk linked to selective reporting bias (Fig. 2).

### Meta-analysis outcomes on upper limb function

**Fig. 3:**
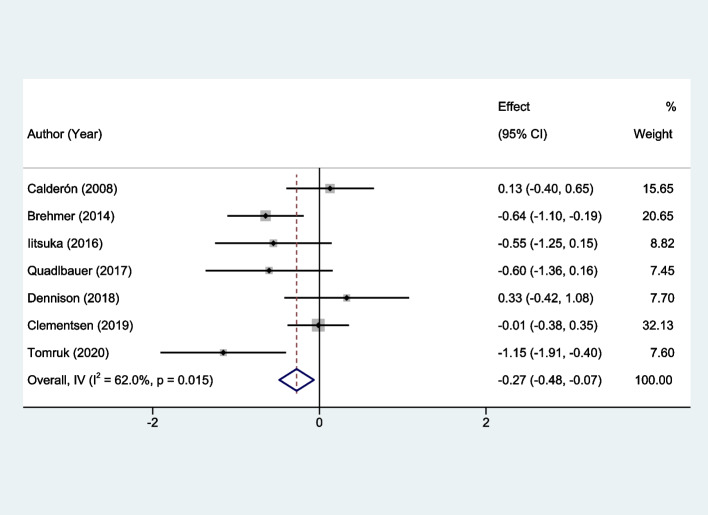
Forest plots of the meta-analysis outcomes on upper limb function

The pooled analysis revealed a statistically significant advantage for the early intervention group over the control group at the 3-month follow-up. Specifically, the standard mean difference (SMD) was -0.27, with a 95% confidence interval (CI) ranging from -0.48 to -0.07 (*P* < 0.0001). The observed heterogeneity was moderate, with an I^2^ value of 62.0% (*P* = 0.015), as illustrated in Fig. 3. The meta-analysis results unequivocally suggest that early rehabilitation is superior to standard care for the restoration of upper limb functionality.

### Meta-analysis findings on wrist function

**Fig. 4:**
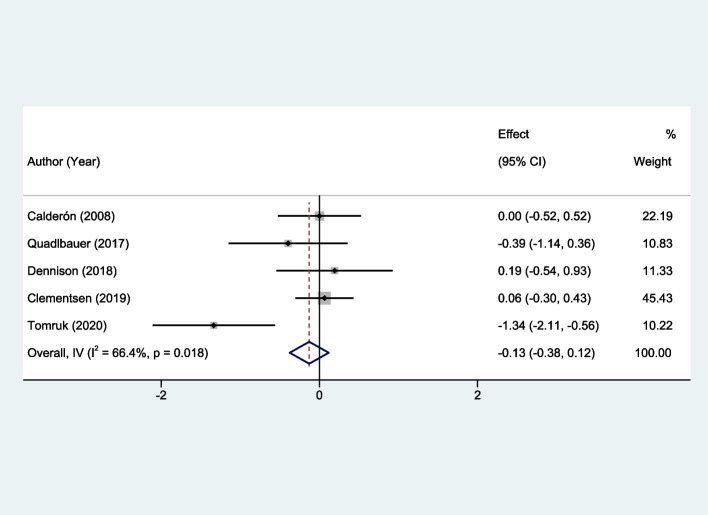
Forest plots of the meta-analysis outcomes on wrist function

For wrist function, the early rehabilitation was also evaluated through corresponding studies with a follow-up duration of 3 months. Although the early intervention group demonstrated better scores compared to the control group, the difference was not statistically significant. The SMD was -0.13, with a 95% CI ranging from -0.38 to 0.12 (*P* = 0.36). Moreover, the heterogeneity was relatively high, with an I^2^ value of 66.4% (*P* = 0.018), as depicted in Fig. 4.

### Meta-analysis findings on back extension mobility

**Fig. 5:**
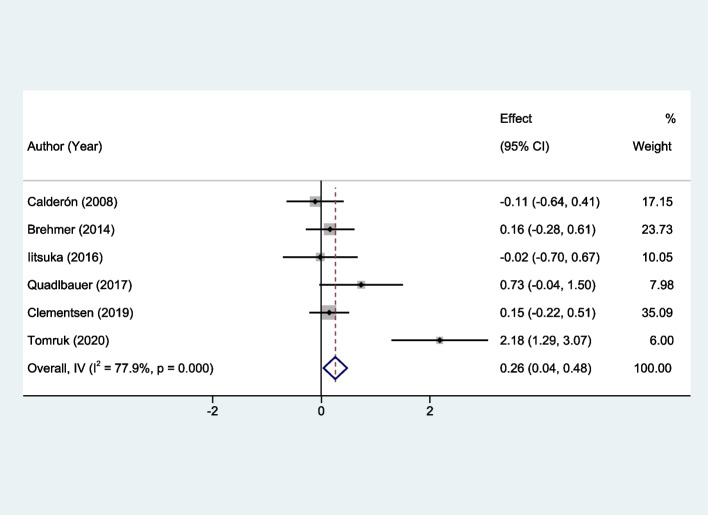
Forest plots of the meta-analysis outcomes on back extension mobility

A statistically significant improvement in back extension mobility was noted in the early rehabilitation group when compared to the control group. The calculated SMD was 0.26, with a 95% CI of 0.04 to 0.48 (*P* = 0.021), as illustrated in Fig. 5.

### Meta-analysis findings on pain

**Fig. 6:**
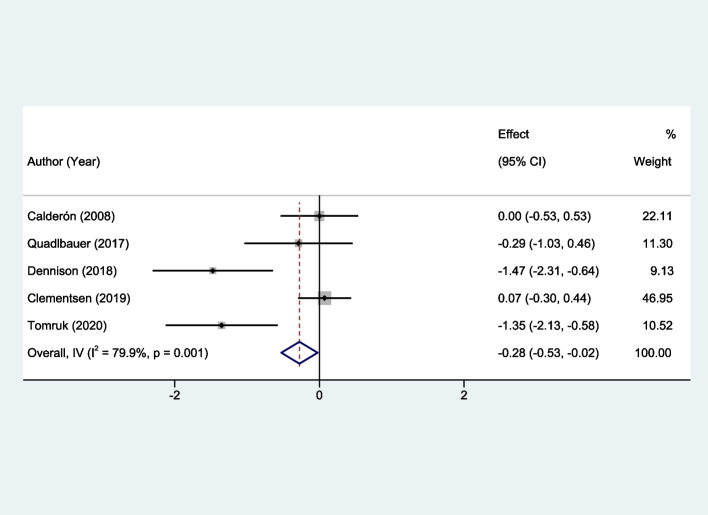
Forest plots of the meta-analysis outcomes on pain

Upon comprehensive statistical analysis, a significant decrease in the pain level was observed in the early rehabilitation group as compared to the control group. The SMD was calculated to be -0.28, with a 95% CI ranging from -0.53 to -0.02 (*P* = 0.03). Notably, the analysis disclosed high heterogeneity among the included studies, with an I^2^ value of 79.9% (*P* = 0.001), as delineated in Fig. 6.

### Meta-analysis findings on complications

**Fig. 7:**
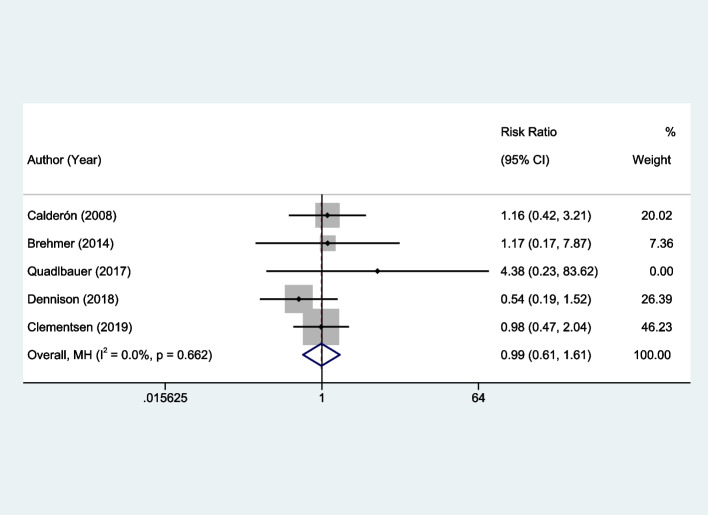
Forest plots of the meta-analysis outcomes on complications

The overall analysis did not show a statistically significant difference between the early rehabilitation and control groups in terms of complications. The OR was calculated as 0.99, with a 95% CI of 0.61 to 1.61 (*P* = 0.96). Remarkably, no heterogeneity was identified across the selected studies, evidenced by an I^2^ value of 0% (*P* = 0.662), as indicated in Fig. 7.

### Publication bias

**Fig. 8:**
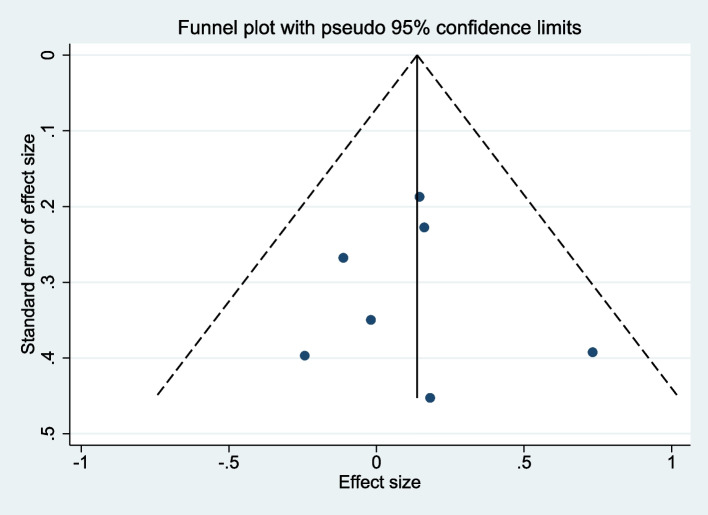
Funnel plot for publication bias in all included studies

In the present meta-analysis, the funnel plots generated for the encompassed studies demonstrated symmetrical distributions, thereby providing no evidence of substantial publication bias (Fig. 8). Further statistical validation using Egger's linear regression test corroborated the absence of significant publication bias across various analyzed variables, with all *p*-values exceeding 0.05. These findings lend additional credence to the integrity and robustness of the meta-analytic outcomes presented herein.

## Discussion

Our meta-analysis, adhering to PRISMA guidelines, conducted an extensive search across key databases, ensuring an inclusive review of distal radius fracture rehabilitation. We highlighted the significant benefits of early rehabilitation on upper limb function and back extension mobility, demonstrating efficacy with minimal heterogeneity. This underscores the holistic benefits of addressing secondary impairments due to immobilization, like altered posture. Despite variability in wrist function improvement and pain management, our findings offer a nuanced view of early rehabilitation's advantages. Our work introduces a comprehensive evaluation of rehabilitation's broad benefits, advocating for tailored strategies to enhance recovery and quality of life. This rigorous approach and detailed analysis underscore the importance of early intervention, contributing valuable insights to the field and setting a foundation for future research.

The distal radius is characterized by its expanded distal end and a rectangular cross-section composed of cancellous bone, making it susceptible to fractures. DRFs can usher in a plethora of challenges that transcend beyond mere skeletal defects [14]. The functional aftermath often manifests as wrist dysfunction, which may involve limited range of motion, stiffness, and loss of strength. Adhesive contracture, a condition characterized by tissue fibrosis leading to restricted joint mobility, is another common sequela of DRFs [15, 16]. These functional limitations are frequently accompanied by pain, both acute and chronic, that can substantially impair the patient's overall quality of life. The complicated clinical picture post-DRF is further marred by the increased incidence of a set of associated complications [17]. These include but are not limited to CTS, a neuropathic disorder resulting from median nerve compression; complex regional pain syndrome (CRPS), a multifactorial disorder characterized by severe localized pain; and tenosynovitis, an inflammation of the synovial sheath surrounding a tendon [18, 19]. Recognizing the intricate complications that accompany DRFs, the American Academy of Orthopaedic Surgeons (AAOS) published comprehensive guidelines in 2020 to address these challenges [18]. These guidelines stipulate that surgical intervention is strongly recommended for unstable DRFs, defined by specific radiological parameters and clinical presentation. Following a stable surgical intervention, the guidelines explicitly advocate for the initiation of early rehabilitative exercises. The study aims to aggregate and critically evaluate existing evidence to yield a more robust understanding of how early intervention through rehabilitation impacts variables like wrist functionality, range of motion, pain levels, and subsequent complications [20, 21].

The meta-analysis comprehensively evaluates the impact of early rehabilitation on functional recovery post-distal radius fracture, showcasing significant benefits in upper limb function (SMD: -0.27; 95% CI: -0.48 to -0.07) and back extension mobility (SMD: 0.26; 95% CI: 0.04 to 0.48), with moderate to no heterogeneity across studies (I^2^: 62.0% and 0%, respectively). These findings highlight early rehabilitation's role in enhancing upper limb functionality and spinal mobility, indicating a holistic improvement in patients' physical health and quality of life. Specifically, the inclusion of spinal extension mobility addresses the broader effects of immobilization post-fracture, such as altered posture and decreased activity, which can lead to secondary impairments. By facilitating early movement, rehabilitation interventions help mitigate these secondary effects, underscoring the importance of a comprehensive approach to recovery. Despite the significant reduction in pain levels observed (SMD: -0.28; 95% CI: -0.53 to -0.02), variability in study outcomes (I^2^: 79.9%) suggests the need for tailored rehabilitation strategies. This analysis, supported by consistent findings and minimal publication bias, reinforces early rehabilitation's efficacy in postoperative care protocols.

Conversely, the findings related to wrist function did not show a statistically significant improvement with early rehabilitation. Although the intervention group fared better in performance scores, the observed differences were not sufficient to attain statistical significance, indicated by an SMD of -0.13 and a 95% CI that straddles zero. Additionally, the relatively high heterogeneity (I^2^ = 66.4%, *P* = 0.018) may suggest that other variables or confounders might have influenced these outcomes. Furthermore, in terms of complications, the meta-analysis found no significant difference between the early rehabilitation and control groups, thereby suggesting that early intervention does not necessarily increase or decrease the risk of adverse outcomes. The lack of heterogeneity in this domain (I^2^ = 0%, *P* = 0.662) points to consistent findings across the studies and therefore lends weight to the validity of this result. In summation, while early intervention appears to be highly beneficial in certain domains such as upper limb function, back mobility, and pain management, its efficacy is less certain in others like wrist function and complication rates. These findings underscore the need for targeted application of early intervention techniques and highlight areas for further research to elucidate the observed heterogeneity.

This meta-analysis presents several limitations that warrant cautious interpretation of the findings. Firstly, the included studies exhibit considerable heterogeneity in both methodology and patient demographics, which could compromise the reliability of our pooled results. Secondly, the short follow-up periods in many of the studies hinder assessments of long-term effectiveness. Additionally, the lack of standardization in rehabilitation protocols and variable quality among the included studies may weaken the conclusiveness of our results.

## Conclusions

In conclusion, early rehabilitation after distal radius fracture surgery is clinically significant. Our meta-analysis shows that early intervention improves upper limb and wrist function, reduces pain, shortens recovery time, and enhances overall quality of life. Thus, it is a crucial component in standardizing patient care post-surgery.

## Data Availability

The datasets used and/or analyzed during the present study are available from the corresponding author on reasonable request.
